# 0032. Relationship between microcirculatory alterations and venous-to-arterial carbon dioxide differences in patients with septic shock

**DOI:** 10.1186/2197-425X-2-S1-O5

**Published:** 2014-09-26

**Authors:** GA Ospina-Tascón, DF Bautista, M Umaña, WF Bermúdez, JD Valencia, HJ Madriñan, A Bruhn, G Hernandez, M Granados, CA Arango-Dávila, D De Backer

**Affiliations:** Fundación Valle del Lili, Universidad ICESI, Intensive Care Medicine Department, Cali, Colombia; Pontificia Universidad Católica de Chile, Facultad de Medicina, Departamento de Medicina Intensiva, Santiago, Chile; Free University of Brussels, Erasme Hospital, Intensive Care Medicine Department, Brussels, Belgium

## Introduction

Increased venous to arterial carbon dioxide difference (Pv-aCO_2_) have been attributed to low cardiac output states. However, mechanisms conducting to Pv-aCO_2_ increases during normal or even high cardiac output conditions as in septic shock are not fully understood. We hypothesized that Pv-aCO_2_ could reflect the adequacy of microvascular perfusion during resuscitated septic shock

## Objectives

To test the hypothesis that Pv-aCO_2_ could reflect the microvascular blood flow during the early phases of resuscitation in septic shock

## Methods

We included 80 patients with a first episode of septic shock admitted to a mixed ICU in a University Hospital over a 12-month period. Time 0 (T0) was set at ICU admission when a pulmonary artery catheter was inserted. Arterial and venous gases analyses were performed at T0 and 6 hours after (T6). We defined Pv-aCO_2_ as the difference between the mixed venous and arterial CO_2_ partial pressures. A Sidestream Dark-Field (SDF) imaging device (Microvision Medical, Amsterdam, the Netherlands) was used to evaluate the sublingual microcirculation both at T0 and T6. At each assessment, 5 sequences of 20 seconds each were recorded and stored under a random number. An investigator blinded to the sequence order and patient's clinical course, analyzed the sequences semi-quantitatively. The vessels were separated into large and small using a cut-off value of 20 µm in diameter. We evaluated the relation between the percentage of small vessels perfused and the Pv-aCO_2_ using linear and non-linear regressions and Spearman Rho test. A p< 0.05 was considered as significant.

## Results

We found significant but very weak relationships between general hemodynamics or oxygen derived parameters with Pv-CO_2_.Pv-aCO_2_ was inversely related to the percentage of small vessels perfused both at T0 and T6 (T0: R^2^:0.515, p< 0.001; T6: R^2^:0.453, p< 0.001).

**Fig. 1:**
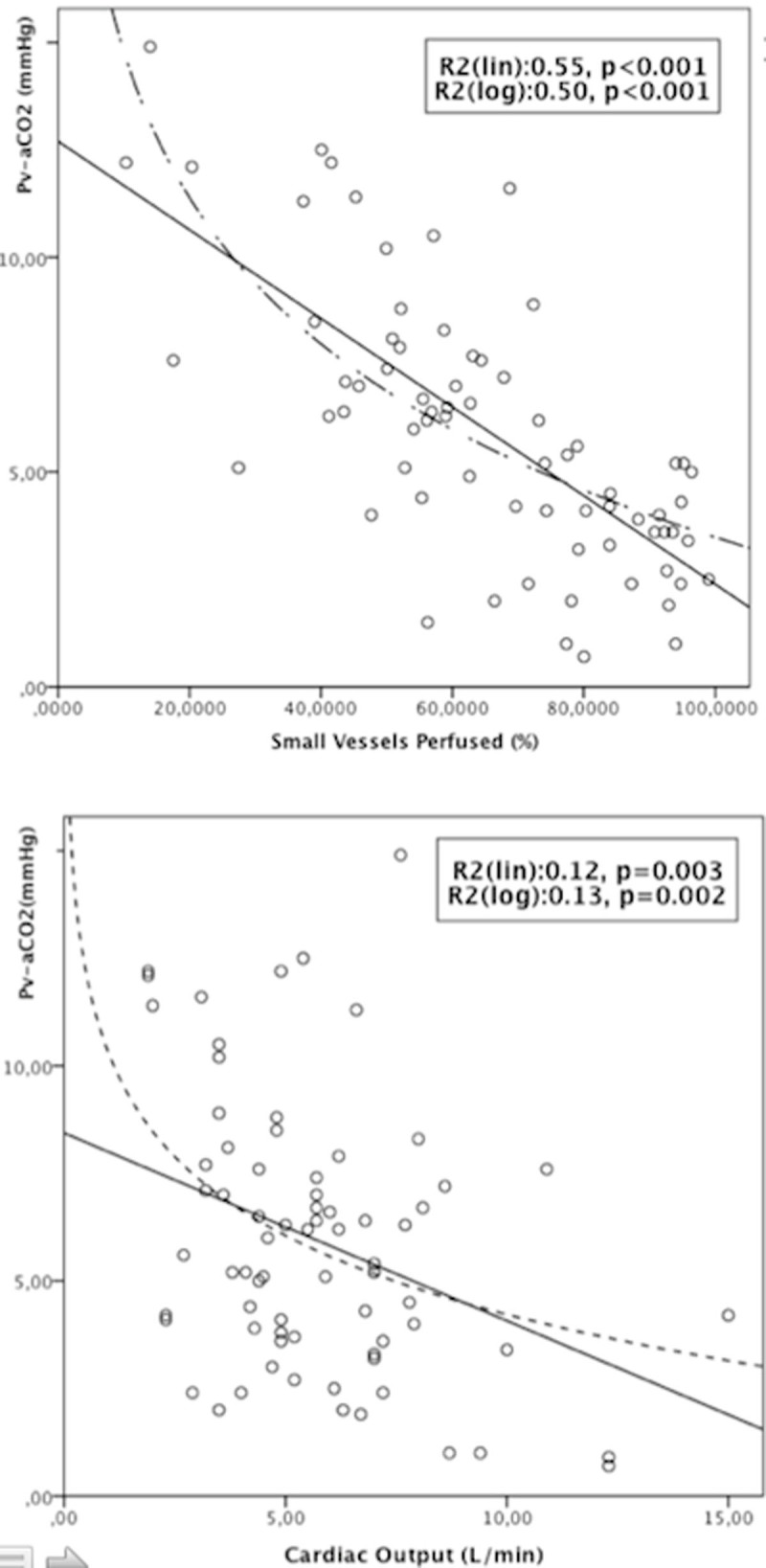
Pv-aCO2 vs. SVP and CO at T0

**Fig. 2:**
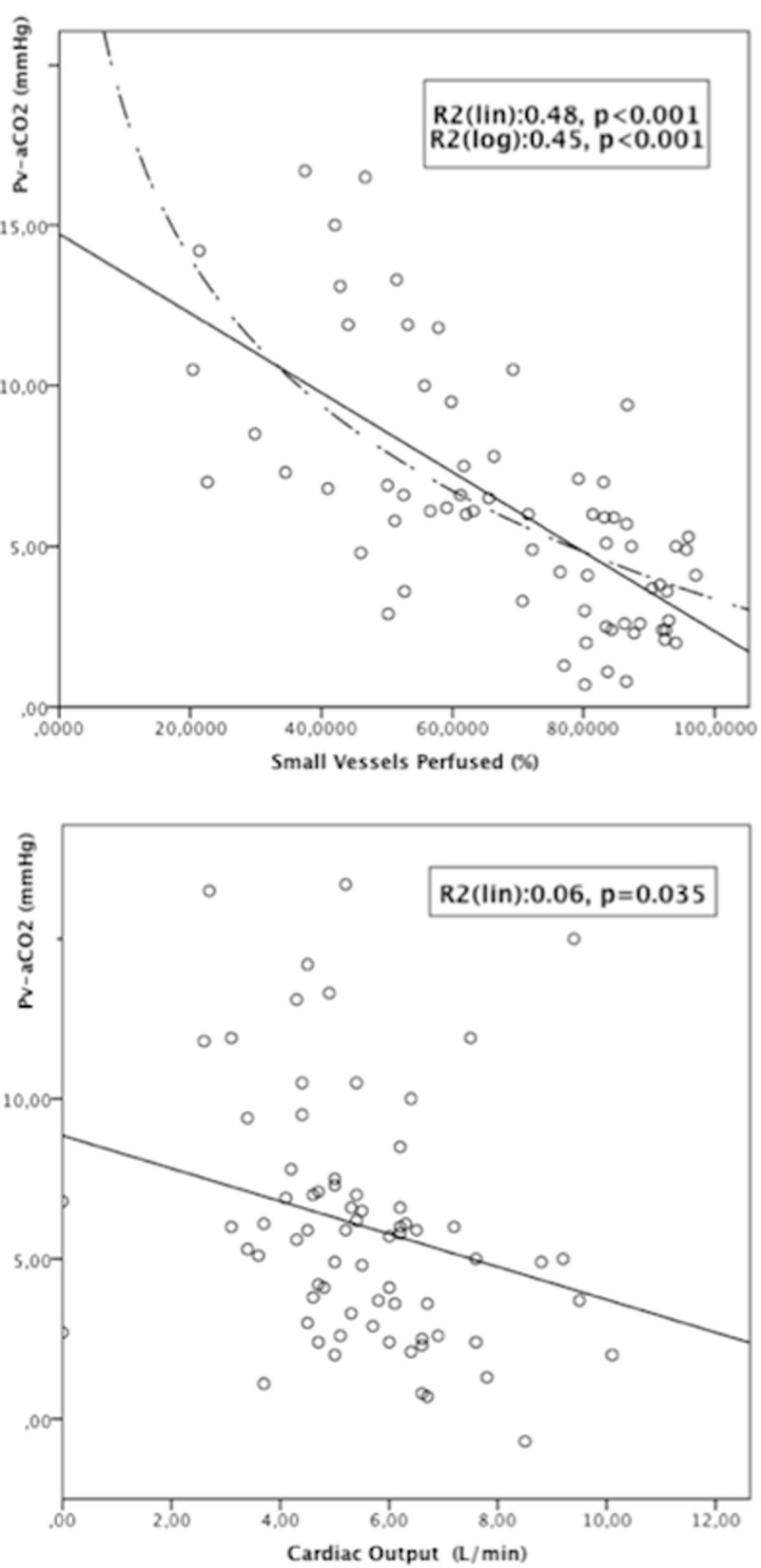
Pv-aCO2 vs. SVP and CO at T6

**Table 1:** General hemodynamics and Oxygen-derived parameters

	T0	T6
SvO2, (%)	68.8 (61.75-75.0)	69.7 (64.4-75.9)
Cardiac Index, (L/min/m2)	3.3 (2.4-4.0)	3.2 (2.7-3.8)
iDO2, (ml/min/m2)	389.0 (293.4-500.3)	399.0(322.3-468.2)
iVO2, (ml/min/m2)	116.8 (87.3-150.4)	182.8 (106.3-240.4)
Pv-aCO2, (mmHg)	5.2 (3.6-7.2)	5.8 (3.5-7.7)

## Conclusions

Microvascular blood flow is a key determinant of Pv-aCO_2_ during normodynamic septic shock. Pv-aCO_2_ could track microvascular alterations during early phases of septic shock.

